# Polo-like Kinase 1 as a potential therapeutic target in Diffuse Intrinsic Pontine Glioma

**DOI:** 10.1186/s12885-016-2690-6

**Published:** 2016-08-18

**Authors:** Vladimir Amani, Eric W Prince, Irina Alimova, Ilango Balakrishnan, Diane Birks, Andrew M. Donson, Peter Harris, Jean M. Mulcahy Levy, Michael Handler, Nicholas K. Foreman, Sujatha Venkataraman, Rajeev Vibhakar

**Affiliations:** 1 Department of Pediatrics, University of Colorado Denver, Anschutz Medical Campus, 12800 19th Ave, Aurora, CO 80045 USA; 2 Department of Neurosurgery, University of Colorado, Anschutz Medical Campus, 12800 19th Ave, Aurora, CO 80045 USA; 3 Children’s Hospital Colorado, 13123 E 16th Ave, Aurora, CO 80045 USA

**Keywords:** DIPG, PLK1, BI6727

## Abstract

**Background:**

Diffuse intrinsic pontine gliomas (DIPGs) are highly aggressive, fatal, childhood tumors that arise in the brainstem. DIPGs have no effective treatment, and their location and diffuse nature render them inoperable. Radiation therapy remains the only standard of care for this devastating disease. New therapeutic targets are needed to develop novel therapy for DIPG.

**Methods:**

We examined the expression of PLK1 mRNA in DIPG tumor samples through microarray analysis and found it to be up regulated versus normal pons. Using the DIPG tumor cells, we inhibited PLK1 using a clinically relevant specific inhibitor BI 6727 and evaluated the effects on, proliferation, apoptosis, induction of DNA damage and radio sensitization of the DIPG tumor cells.

**Results:**

Treatment of DIPG cell lines with BI 6727, a new generation, highly selective inhibitor of PLK1, resulted in decreased cell proliferation and a marked increase in cellular apoptosis. Cell cycle analysis showed a significant arrest in G2-M phase and a substantial increase in cell death. Treatment also resulted in an increased γH2AX expression, indicating induction of DNA damage. PLK1 inhibition resulted in radiosensitization of DIPG cells.

**Conclusion:**

These findings suggest that targeting PLK1 with small-molecule inhibitors, in combination with radiation therapy, will hold a novel strategy in the treatment of DIPG that warrants further investigation.

## Background

Diffuse intrinsic pontine glioma (DIPG) is a childhood brain tumor that is largely fatal with median survival time of just 9–12 months and accounts for the majority of pediatric brain tumor mortality [1, 2]. These tumors are highly infiltrative within the ventral pons that render them inoperable. There are no effective chemotherapeutic options available for treatment of these devastating tumors. Radiation therapy remains the standard of care [2]. The shortage of targeted therapy can be attributed to the lack of understanding the biology associated with DIPG. Until recently, a shortage of primary tissue available for research limited the understanding of the genomic landscape of DIPG. With new biopsy protocols in place, and a growing number of cell lines that create robust preclinical models, experimental models of DIPG have been established to investigate the biology of the disease [3].

These experimental models have provided new insight into the role of the genetic makeup of DIPG, potential drivers of this tumor, as well as potential therapeutic targets. Initial whole-genome profiling of DIPG identified recurrent involvement of platelet-derived growth factor receptor alpha (PDGFRA) pathways [4]. Follow up studies with larger sample sets supported this finding, as well as amplification of genes within other tyrosine kinase-Ras-phophoinositide 3-kinase signaling pathways such as MET. In addition, a portion of DIPG had amplification of cell-cycle regulatory genes for retinoblastoma protein (RB) phosphorylation [5]. Additional advances using whole genome exome sequencing demonstrated the presence of mutations in histone H3F3A or H3.1 (lysine 27 > Methionine, K27M) in a high proportion of DIPG tumors [6]. Further these histone mutations have potential biologic and clinical relevance in patients. For example the presence of the K27M-H3.3 mutation is associated with shorter survival [7]. Additional studies have identified mutations in ACVR1, and ATRX [8–10]. Studies also suggest that DNA Damage response pathways are altered in DIPG, perhaps reflecting the fact that these tumors are highly radio-resistant [4, 11]. We hypothesized that kinases involved in response to DNA damage and radiation sensitization would be attractive targets for DIPG therapy. We chose to initially examine polo-like kinase 1 (PLK1) based on our previous experience with this kinase as a radio-sensitizer in another brain tumor, medulloblastoma.

PLK1 is essential for mitotic progression, regulating entry into mitosis by phosphorylating cyclin B1/CDK1 complex, and exit by activating the Anaphase Promoting Complex (APC) [12]. Non-mitotic roles of PLK1 have also been described in the form of anti-apoptotic function [13, 14]. PLK1 is overexpressed in a variety of human cancers, and overexpression is linked to chromosomal instability and aneuploidy [12]. PLK1 may play an oncogenic role in tumor survival and growth, as inhibition of PLK1 by shRNA or small molecule inhibitors has been shown to decrease cell proliferation both in vitro and in vivo [12, 15, 16]. In fact, forced expression of PLK1 in human fibroblasts in vitro, is capable of creating xenograft tumors in nude mice [17]. Importantly, small molecule inhibition resulted in lower proliferation of cancer cells versus normal cells [18, 19]. Phase I/II trials of PLK1 inhibitors in advanced solid tumors in adults have yielded promising results [20, 21]. For these reasons, small-molecule inhibitors of PLK1 have become attractive candidates for drug development.

We and others have identified PLK1 as a potential therapeutic target in brain tumors including glioblastoma and medulloblastoma [22–24]. Importantly PLK1 inhibition strongly radio-sensitized medulloblastoma cells [22]. PLK1 has been identified as a potential therapeutic target in other pediatric tumors such as rhabdomyosarcoma and neuroblastoma as well [25–27]. Volasertib (BI 6727) is a new generation, highly selective PLK1 inhibitor that acts as an ATP-competitive kinase inhibitor of PLK1 [28]. We chose to use this inhibitor to examine the effects of PLK1 inhibition in DIPG, as it is currently the most clinically advanced of the investigational PLK1 inhibitors [29].

In this study, our goal was to evaluate PLK1 as a potential therapeutic target in DIPG. We show that PLK1 is abnormally over expressed in DIPG compared to normal pons and that inhibition of PLK1 significantly impaired DIPG cell growth and induced DNA damage in vitro. We further show that PLK1 inhibition with clinically relevant inhibitors can radio-sensitize DIPG cells.

## Methods

### Cell lines and primary patient samples

DIPG IV and VI cells were kindly provided by Dr. Michelle Monje (Stanford University, California) and cultured in tumor stem media (TSM) consisting of Neurobasal(-A) (Invitrogen), B27(-A) (Invitrogen), human-basic FGF (20 ng/mL; Shenandoah Biotech), human-EGF (20 ng/mL; Shenandoah Biotech), human PDGF-AB (20 ng/mL; Shenandoah Biotech) and heparin (10 ng/mL). Both DIPG IV and DIPG VI cells carry the H3K27M mutation and DIPGVI cells also harbor an additional TP53 point mutation [30].

Primary patient samples were obtained from Children’s Hospital Colorado and were conducted in accordance with local and federal human research protection guidelines and Institutional Review Board (IRB) regulations. Informed consent was obtained for all specimens collected. Normal brain tissue was collected from autopsy at the Children’s Hospital Colorado under IRB guidelines.

### Gene expression microarray analysis

Eight patient tumor samples were evaluated for gene expression using Affymetrix U133 Plus 2.0 GeneChip microarrays. Briefly, samples were snap-frozen in liquid nitrogen at the time of surgery. An RNeasy kit (Qiagen, Valencia, CA) was used to extract ribonucleic acid from each sample using. Samples were hybridized to HG-U133 Plus 2.0 GeneChips (Affymetrix, Santa Clara, CA) according to the manufacturer’s instructions. All microarray data from the samples was background-corrected and normalized using the gcRMA algorithm. One probe set per gene was selected for use in subsequent analyses based on highest overall expression level across samples. Differential expression of genes was determined using a Student’s *t*-test.

### Small molecule inhibitors of PLK1

The small molecule PLK1 inhibitor BI 6727 was purchased from Selleck Chem (Houston, TX). The drug was reconstituted in dimethyl sulfoxide (DMSO) and stored according to manufacturer’s instructions. An equivalent amount of DMSO for the highest concentration of drug was used for each experiment as a vehicle control.

### Cell proliferation and apoptosis

Cell number and viability was determined using Viacount assays. 5,000 cells were plated into ultra-low attachment U-bottom 96 well plates (Corning) and treated 24 h later with appropriate doses of BI 6727 for 72 h. Cells were pelleted, dissociated using TrypLE Express (Invitrogen) and stained with ViaCount reagent (Millipore, Billerica, MA). Samples were run on a Guava EasyCyte Plus flow cytometer (Millipore).

Cell proliferation was also determined by MTS [3-(4, 5-dimethylthiazol-2-yl)-5-(3-carboxymethoxyphenyl)-2-(4-sulfophenyl)-2H-tetrazolium] assay using CellTiter 96 AQueous One Solution (Promega, Madison, WI). For phamracologic experiments, cells were plated and treated 24 h later with increasing doses of BI 6727 for 72 h. MTS reagent was added according to standard manufactures protocol. Plates were read using a BioTek Synergy 2 plate reader (Winooski, VT). Experiments were done in triplicate and background absorbance was subtracted from all wells before analysis.

Apoptosis was assessed 72 h after BI 6727 treatment using Guava Nexin reagent (Millipore). Samples were run on a Guava EasyCyte Plus flow cytometer (Millipore). All treatments were run in triplicate. GraphPad Prism 5 software was used to analyze the results.

### Western blotting

Protein lysates were obtained from samples using RIPA buffer (Thermo Scientific, Rockford, IL) with protease inhibitors added. Western blotting was performed per standard methods. Antibodies for PLK1 (#4535), Rad51 (#8875S), phospho γH2AX (2577), p21(2947), p53(calbiochem OP03) and Actin (MAB1501) were purchased from Cell Signaling Technology (Danvers, MA) and Millipore, respectively. Secondary antibodies conjugated to horseradish-peroxidase were used in conjunction with a chemiluminescent reagent to visualize protein bands.

### Phospho-histone H2AX (γH2AX) foci immunofluorescent microscopy and imaging

3,000 cell were plated and treated with BI 6727 for 6 and 24 h in poly-D-lysine (Sigma) coated chamber slides. Cells were then washed and fixed with 4 % paraformaldehyde for 15 min at room temperature (RT). Afterwards 0.2 % Triton X-100 in PBS was used to permeabilize cells for 15 min followed by blocking in 5 % milk diluted in 0.05 % Triton X-100 for 30 min at RT. Next, primary antibodies were applied: anti-γH2AX (Ser139) was used at a dilution of 1:200. After several washes, Alexa Fluor 488 conjugated secondary antibody (1:500) was applied for 1 h at room temperature in the dark. ProLong Gold Antifade reagent (Life Technologies) containing DAPI (Life Technologies) was used for mounting. Images were acquired using an inverted epifluorescence microscope at a magnification of 40x (oil). At least three random fields were chosen to count cells containing greater than 10 foci.

### Combination of BI 6727 and ionizing radiation

5,000 cells/well were plated into 96-well plates (Corning) and treated with BI 6727 24 h later. Cells were exposed to drug for 24 h, and then drug-containing medium was aspirated and normal culture medium was added. Cells were immediately irradiated using a Cesium source irradiator. Cells were allowed to grow for 120 h, and MTS assays as above were used to measure proliferation. Survival curves were generated after normalizing for the amount of BI 6727-induced death. Non-linear regressions were calculated for each line. The radiation dose intersecting the non-linear regression for a 10 % (SF0.1) and 50 % (SF0.5) surviving fraction was calculated for each drug dose as previously described (Harris et al.)

### Statistical analysis

Student’s *t*-test was used to calculate any statistical significance. Error bars represent the standard error of the mean (*n* ≥ 3). GraphPad Prism 5 was used to calculate IC_50_ values and to compute the nonlinear regression equations.

## Results

### PLK1 is overexpressed in DIPG

**Fig. 1:**
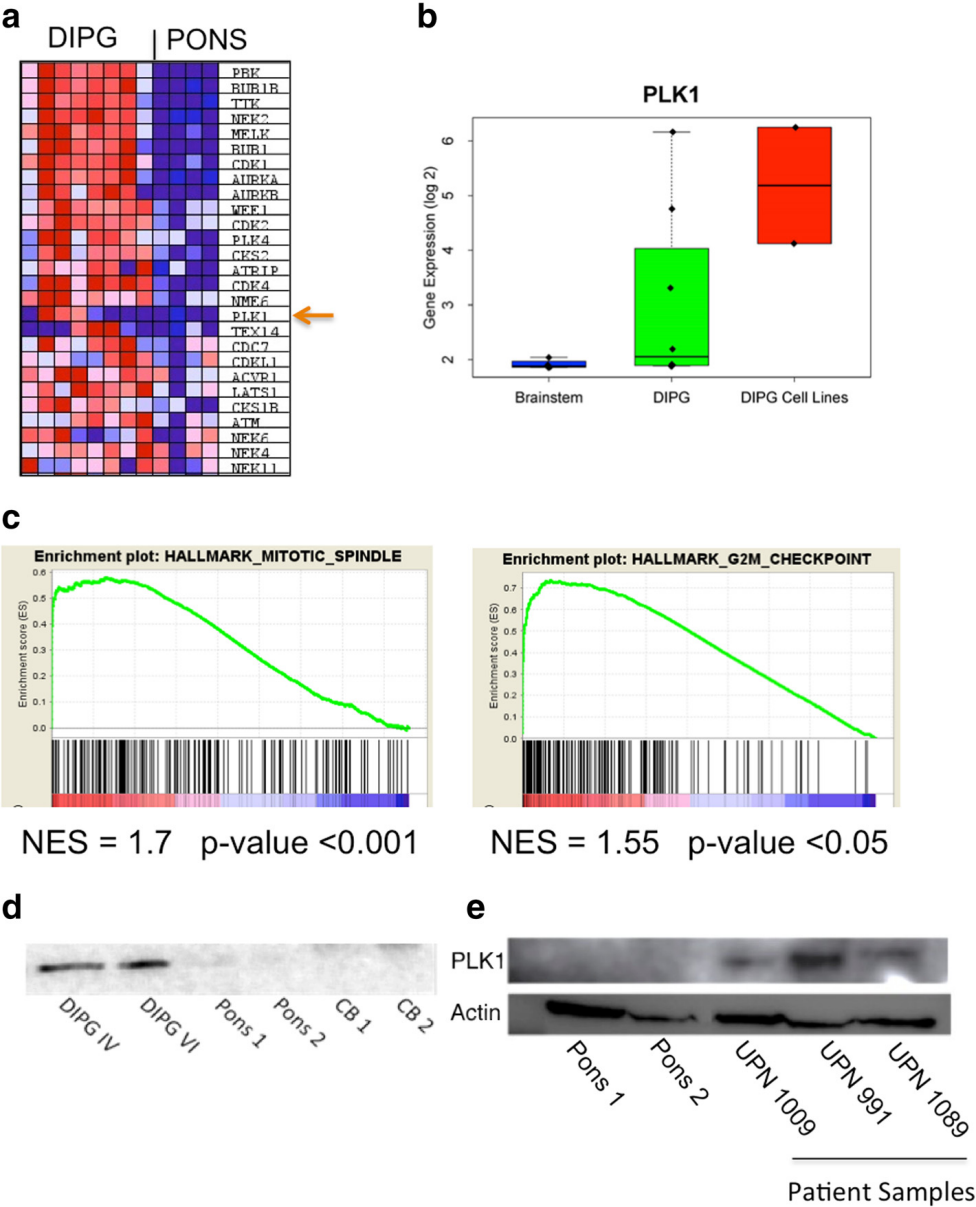
PLK1 is overexpressed in DIPG patient samples and cell lines. **a** Gene expression of a panel of mitotic checkpoint kinases in patients with DIPG versus normal pons. Arrow shows up-regulation of PLK1 in DIPG samples. **b** Microarray data analysis of PLK1 gene expression in brainstem, patient DIPG samples, and DIPG cell lines. Expression level ± SEM is shown (**c**) Gene enrichment analysis of patient DIPG samples compared to normal pons from microarray data for PLK1 associated gene signatures. **d** Confirmation of elevated PLK1 protein expression in DIPG cell lines compared to normal pons and cerebellum (CB) by western blot. **e** Western blot analysis of PLK1 protein in normal pons and three patient samples

Kinases that regulate cell cycle and mitotic progression are attractive candidates for targeted therapy in a variety of human cancers [31] and have been shown to be potentially important in DIPG [5]. In order to assess the significance these kinases play in the context of DIPG, we analyzed eight patient samples to determine the relative expression of a panel of mitotic checkpoint kinases versus normal brain controls. We found mitotic kinases are upregulated in the tumors versus the controls (*p* < 0.01), suggesting that they play an important role in DIPG tumorigenesis (Fig. 1a). PLK1, a target with available small molecule inhibitors, was one of the 20 most significantly overexpressed kinases in DIPG compared to normal pons (Fig. 1a). PLK1 transcript was significantly elevated in both patient samples and DIPG cell lines compared to normal brainstem tissue (*p* < 0.01, Fig. 1b). Gene enrichment analysis of primary tumor microarray data demonstrated PLK1 associated gene signatures including the G2M checkpoint (NES = 1.55, *p* < 0.05) and mitotic spindle assembly (NES = 1.7, *p* < 0.05) were also higher in DIPG tumors compared to normal pons (Fig. 1c). Elevated PLK1 protein levels (compared to normal brain) were also identified in two DIPG cell lines DIPG IV and DIPG VI indicating these cells are a good in vitro experimental model to evaluate PLK1 inhibition response (Fig. 1d). Moreover PLK1 protein is significantly elevated in patient samples as shown in Fig. 1e further corroborating the mRNA data suggesting that PLK1 is a potential therapeutic target in DIPG.

### Inhibition of PLK1 suppresses DIPG cell growth and proliferation

**Fig. 2:**
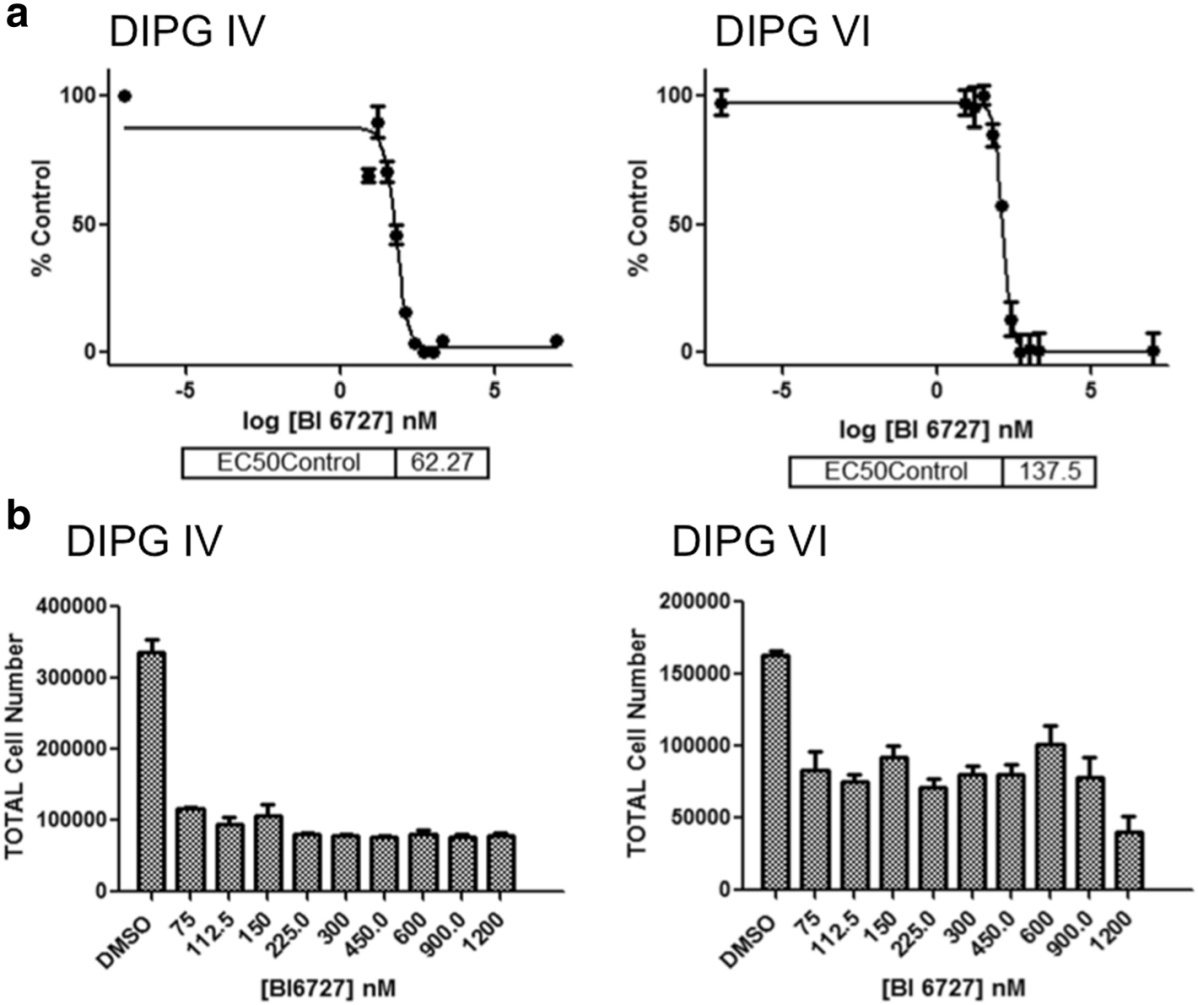
Small molecule inhibition of PLK1 BI6727 suppresses in vitro DIPG cell proliferation and growth. **a** DIPG cells were treated for 72 h with increasing dose of the PLK1 small molecule inhibitor BI 6727. Cell proliferation was measured by MTS assay with the mean ± SEM shown. IC_50_ (in nM) values were calculated as shown. **b** DIPG cells were treated with increasing doses of BI 6727 for 72 h and total viable cell number was measured (± SEM) using Guava flow cytometry

To examine whether PLK1 was functionally important for DIPG tumorigenesis, we treated DIPG cell lines with BI 6727 and noted changes in cell proliferation. To confirm the effect of BI 6727 on cell proliferation, we used MTS assays to establish drug IC50 values. Both cell lines showed a dose dependent response to BI 6727. DIPG IV cells showed greater than two-fold the sensitivity to BI 6727 compared to DIPG VI cells with a 72 h IC_50_ of 62.3 nM versus DIPG VI with an IC50 of 137.5 nM (Fig. 2a). These data might be reflective of the additional TP53 mutation in DIPG VI cells. Guava ViaCount assays performed on neurospheres 72 h after treatment with increasing doses of BI 6727 indicated that both DIPG IV and VI showed marked decrease in cell number versus DMSO controls (Fig. 2b). Interestingly there is a maximum biological effect at 75nM BI 6727 after which there is no further dose dependence.

### PLK1 inhibition induces G2-M arrest and apoptosis in DIPG cells

**Fig. 3:**
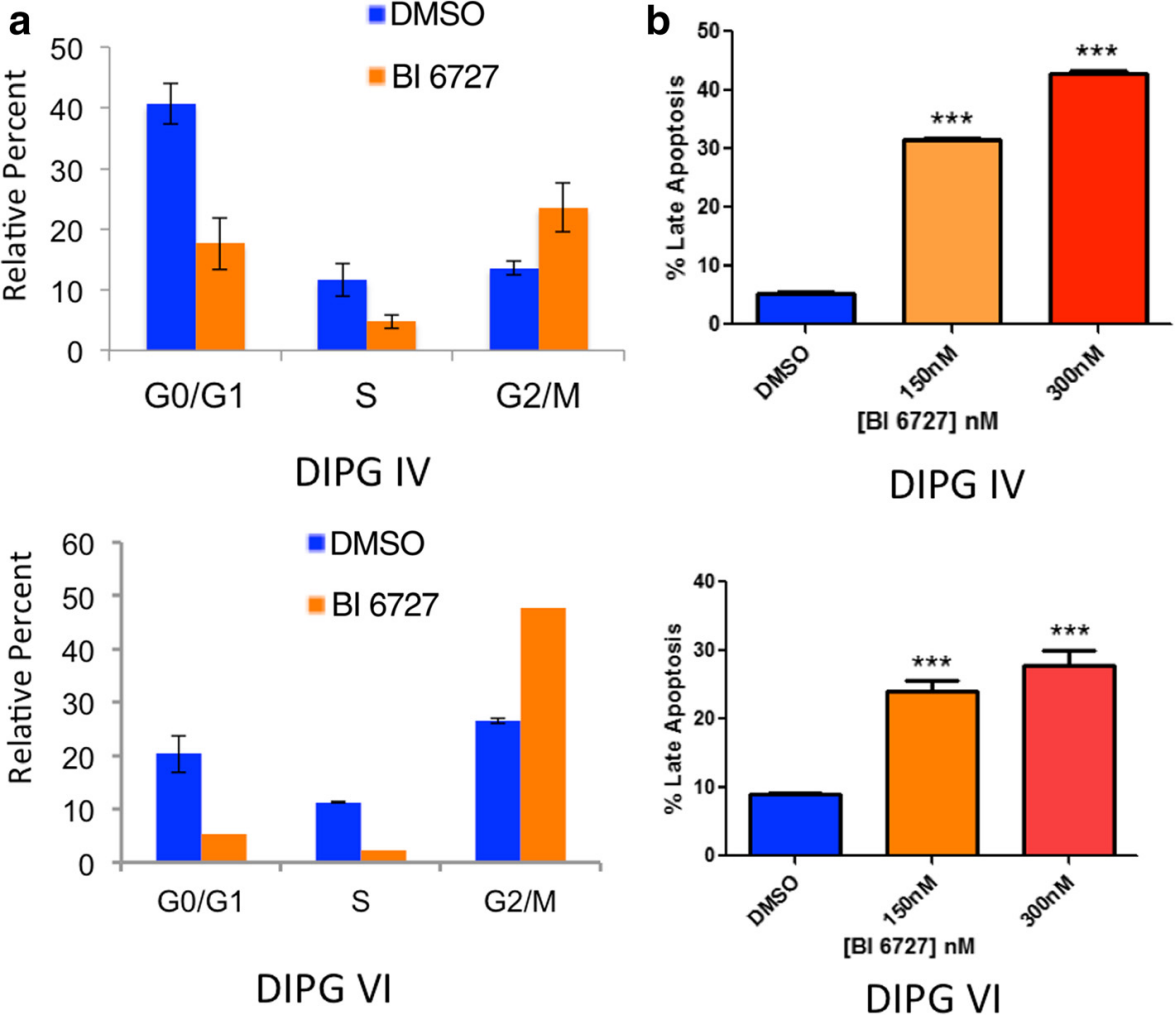
Inhibition of PLK1 induces G2-M arrest and apoptosis in DIPG tumor cells. **a** DIPG cells were treated with IC_50_ doses of BI 6727 for 24 h and analyzed for changes in cell cycle by flow cytometry. **b** DIPG cells were treated with varying concentrations of BI 6727 for 72 h and analyzed for Annexin V/7-AAD positive late apoptosis markers by flow cytometry. The percent of cells in late apoptosis ± SEM is shown

PLK1 is a cell cycle kinase that regulates mitosis by acting as an early trigger for G2-M transition. In order to evaluate the effect of BI 6727 on the cell cycle, we treated DIPG IV and VI cells with their corresponding IC_50_ doses of BI 6727 (Fig. 2a) for 24 h. Cell were fixed and stained, and significant changes cell cycle were observed. Most notably, there was a significant G2-M arrest in BI 6727 treated samples (Fig. 3a). In order to evaluate whether PLK1 inhibition resulted in apoptosis in DIPG cells, we treated DIPG cells with 150 nM and 300 nM of BI 6727 and stained with Annexin V. In each drug-treated cell line, Annexin V positive-7-AAD positive late apoptotic populations were significantly enhanced and Annexin V negative-7-AAD negative live cell populations were subsequently decreased, both in a dose dependent manner (Fig. 3b). The difference in apoptosis between DMSO and BI 6727 treated cells was statistically significant in multiple independent assays.

### BI 6727 treatment induces DNA damage in DIPG cell lines

The histone H3.3 point mutation K27M is a hallmark of DIPG, and abnormal H3.3 function at the embryonic level has recently been shown to cause dysfunction of heterochromatin structures leading to mitotic defects and resulting in karyotypical abnormalities and DNA damage [32]. H3.3 K27M may therefore render DIPG unable to maintain genomic integrity. This provides a potential therapeutic window where induction of further DNA damage could result in more catastrophic cellular events leading to selective tumor cell death.

**Fig. 4:**
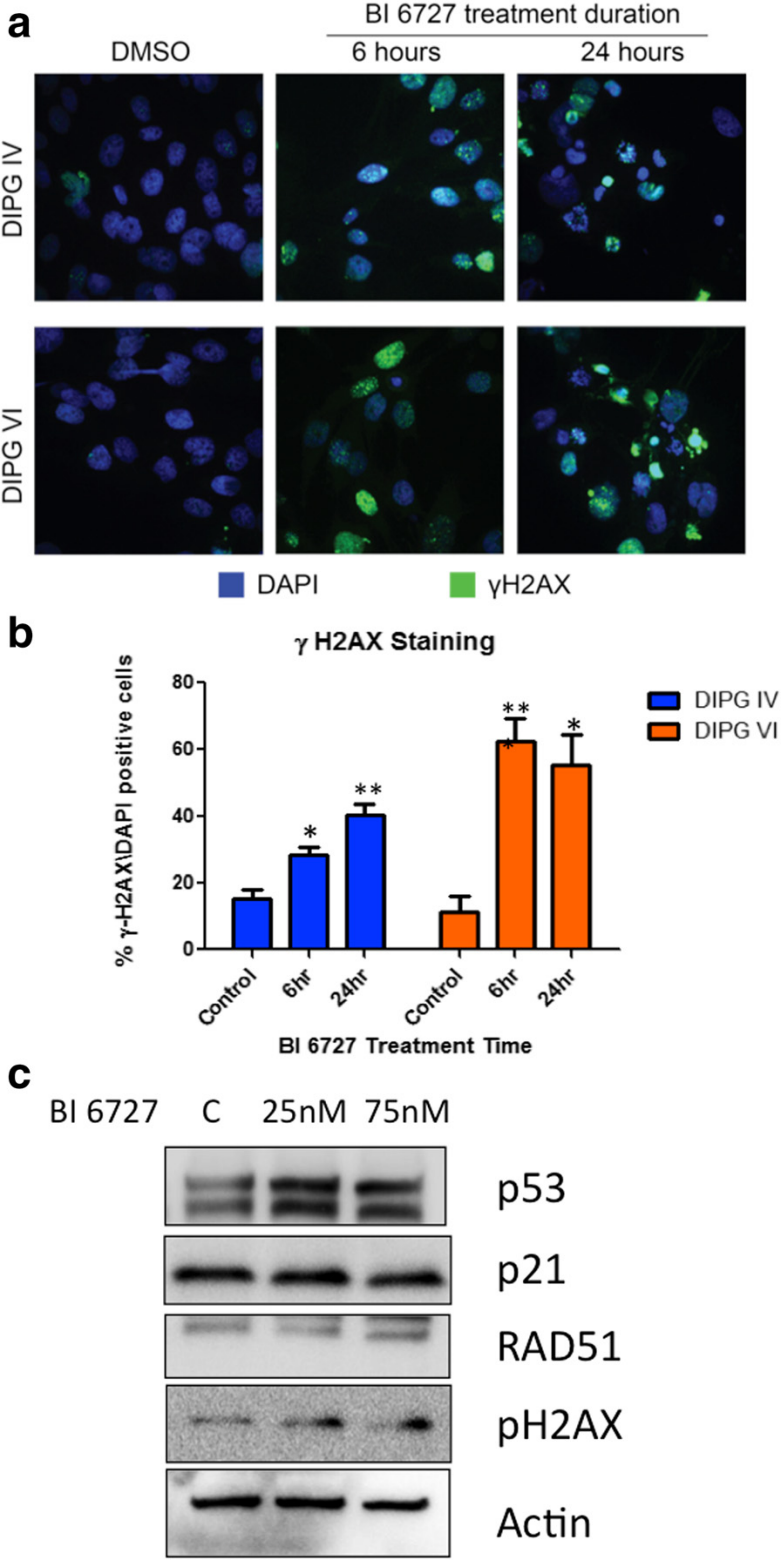
Small molecule inhibition of PLK1 induces DNA damage in DIPG cells. **a** DIPGIV cells were treated with IC_50_ dose of BI 6727 for 6 or 24 h and analyzed for phospho-histone H2AX (γH2AX) by immunofluorescent imaging. Representative images are shown; blue-DAPI nuclei stain and green- γH2AX. **b** Quantification of the percent of γH2AX positive cells identified in (A) normalized to DAPI. Percent ± SEM is shown. **c** DIPG4 cells were treated with IC_25_ and IC_75_ doses of BI 6727 for 24 h and expression of the DNA damage response proteins p53, p21, RAD51, and phospho-γH2AX were evaluated by western blot analysis

To examine the impact of BI 6727 on DNA damage in DIPG cells, we examined the induction of the DNA damage associated marker γH2AX (Fig. 4a). Six hours of treatment with the corresponding IC_50_ dose of BI 6727 induced DNA damage as detected by immunocytochemistry of γH2AX foci in both DIPG IV and DIPG VI cells. As shown in Fig. 4b, the increase in γH2AX foci was statistically significant (*p* < 0.01). A further increase in γH2AX foci was noted in both lines after treatment with BI 6727 for 24 h, indicating an accumulation of DNA damage over time (Fig. 4b).

To further examine induction of DNA damage we evaluated the expression of proteins associated with the Dna Damage Repair (DDR) in response to BI 6727 treatment (Fig. 4c). Treatment with BI 6727 potently induced expression of p53 and γH2AX proteins but not p21, further confirming the induction of DNA damage in response to PLK1 inhibition.

### BI 6727 pretreatment sensitizes DIPG cells to ionizing radiation

**Fig. 5:**
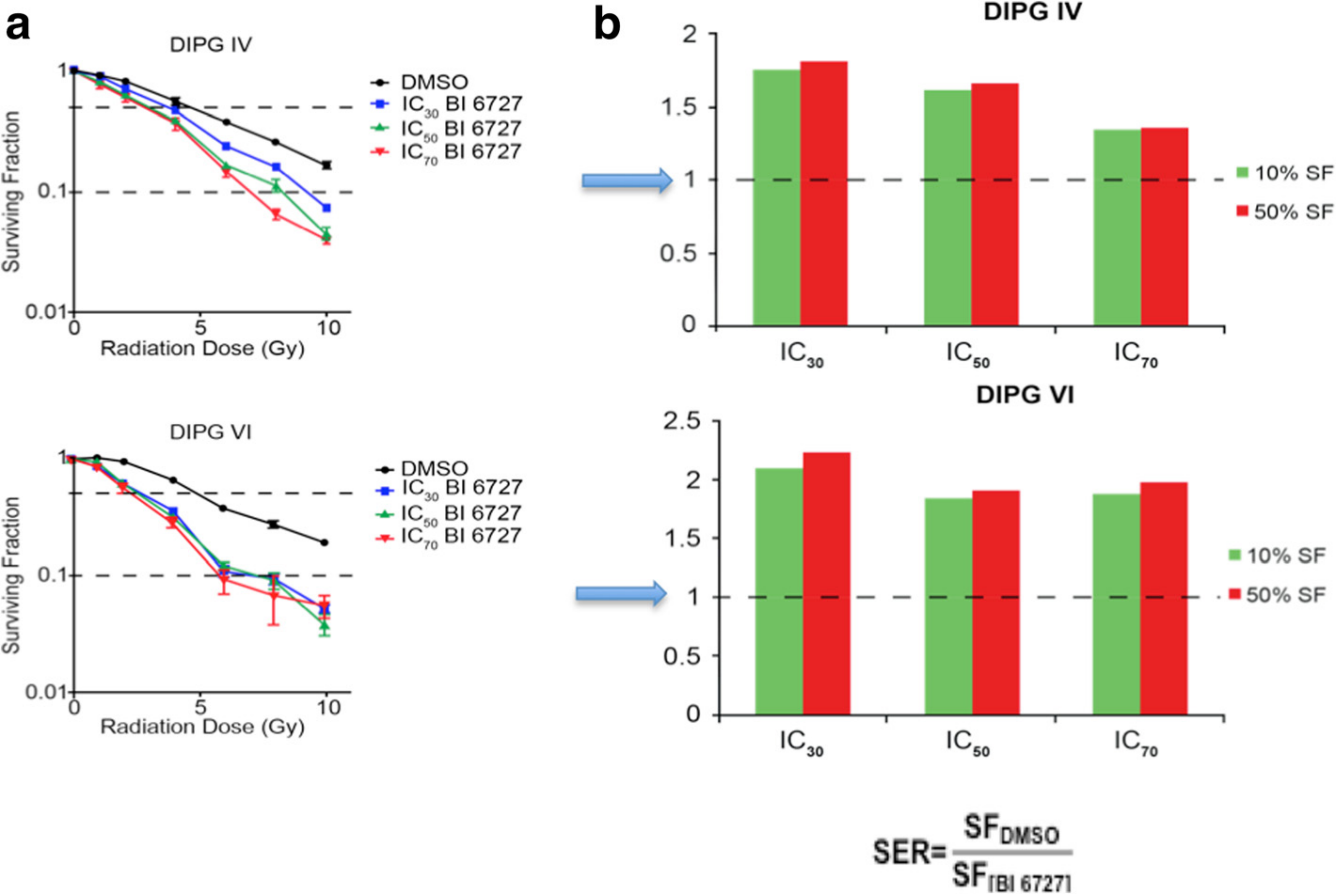
Pre-treatment of DIPG cells with BI 6727 sensitizes them to ionizing radiation. **a** DIPG cells treated with IC_30_, IC_50_ and IC_70_ doses of BI 6727 for 24 h were then exposed to increasing doses of radiation. Cell proliferation was measured by MTS 5 days after radiation. Mean ± SEM shown **b** Calculated sensitizer enhancement ratios (SER) with 24-hour pre-treatment of BI 6727 at IC_30_, IC_50_ and IC_70_ doses

To investigate whether BI 6727 enhances cellular sensitivity to ionizing radiation, DIPG cells were exposed to BI 6727 for 24 h before irradiation, and the effects evaluated using an MTS assay. DIPG IV and VI cells were treated with a DMSO control and the corresponding IC_30_, IC_50_ and IC_70_ doses for each cell line. Drug was washed off, and each plate was exposed to a specific dose of radiation: 0, 1, 2, 4, 6, 8 and 10 Gy. After 5 days of recovery, MTS proliferation assays revealed that the survival fractions (SF) at IC_30_, IC_50_ and IC_70_ doses of BI 6727 were reduced in both DIPG IV and VI cells after exposure to a range of doses of radiation (Fig. 5a). Sensitizer enhancement ratios (SERs) for each cell line were calculated by fitting nonlinear regression curves to the MTS data. For DIPG IV cells, the SERs were 1.7, 1.6 and 1.4 at 10 % cell survival (SF0.1) and 1.8, 1.7 and 1.4 at 50 % cell survival (SF0.5) at IC_30_, IC_50_ and IC_70_ BI 6727 doses respectively. DIPGVI cells also showed increased SERs at 2.1, 1.9 and 1.9 at 10 % cell survival (SF0.1) and 2.2, 1.9 and 2.0 at 50 % cell survival (SF0.5) at IC_30_, IC_50_ and IC_70_ BI 6727 doses, respectively (Fig. 5b). Any SER value greater than one indicates enhancement of radiation. Thus, the radiation survival curves generated establish that BI 6727 pretreatment radiosensitizes DIPG cells to ionizing radiation at a considerable range of doses in both DIPG cell lines.

## Discussion

DIPG remains a therapeutic challenge. Over 200 Phase 1/2 trials have been performed, none of which have shown any promise for therapeutic efficacy [1]. The major problem has been lack of any preclinical data to support these clinical studies. New biopsy protocols have not only allowed for a greater understanding of the biology of the disease through genomic analysis, but also allowed for robust preclinical models to test drugs on targets established from the genomics. Using these newly available cells we show that PLK1 is a novel therapeutic target in DIPG. We demonstrate that PLK1 is significantly up-regulated in DIPGs and that inhibition of PLK1 using a clinically relevant drug, BI 6727 (Volesertib) radiosensitizes DIPG cells across a broad range of doses.

It has been well established that PLK1 is implicated in cell cycle regulation by functioning in centrosome maturation, spindle formation, mitotic entry and cytokinesis [12]. PLK1 overexpression has been linked to invasive phenotypes, highly aggressive tumors and subsequent poor outcomes, all hallmarks of DIPGs [12]. High PLK1 expression has been described in many forms of cancer, yet its role in DIPG tumorigenesis has not previously been explored.

Analysis of mitotic checkpoint kinases revealed a global up-regulation of these kinases in our cohort of DIPG primary tumors. PLK1 emerged as an attractive target, as it is overexpressed in DIPG versus normal pons. Associated spindle assembly and G2-M pathways were also up in DIPGs, confirming that PLK1 function increased with elevated expression. Cell line PLK1 transcript and protein levels were also found to be up-regulated versus normal pons, giving us an ideal model to investigate PLK1 inhibition. The availability of BI 6727, a new generation, highly-selective small molecule inhibitor that is in later phases of clinical development, made it easy to test the effects of PLK1 inhibition in our in vitro model.

Treatment of cells with clinically relevant doses of BI 6727 resulted in a marked reduction of cell proliferation. Post-treatment cell cycle analysis revealed significant G2-M arrest, supporting the idea that combination with radiation would result in enhanced cytotoxicity. Disrupting the proper formation of mitotic spindles required for chromosome alignment and segregation has been shown to preferentially kill cancer cells, and the G2-M phase has been associated with critical DNA repair mechanisms. Indeed we saw significant increase in cell death with BI 6727 treatment alone. Importantly we identified a maximum dose for biological effect after wich dose escalation did not make a difference to cell viability. This observation is important because it suggests that biological effect could be achieved before a maximum tolerated dose in human trials, which might spare patients from toxicity.

We also noted a marked increase in γH2AX staining only 6 h after treatment, and sustained or increased staining after 24 h, indicating an accumulation of DNA damage over time. The accumulation of γH2AX staining and G2-M arrest led us to believe that there was significant chromosomal instability and DNA damage 24 h after BI 6727 treatment in DIPG cells. Indeed we found that irradiation of DIPG cells pre-treated with the varying doses of the BI 6727 potently enhanced the efficacy of ionizing radiation.

More recently Grasso *et al* performed a screen of 83 drugs with therapeutic applications in pediatric oncology [30]. They identified the HDAC inhibitor panobinostat as a promising potential agent for DIPG therapy. This study was the first comprehensive effort to identify therapeutic agents for DIPG in a preclinical model. However, their chemical screen did not include any agents that target PLK1. Other recent studies have identified Aurora Kinase B and CDK4 as additional drugable targets in DIPG [33, 34].

## Conclusions

Together with our data, these and other studies are beginning to use robust pre-clinical cell and animal models to identify exciting new therapeutic options for DIPG. Our findings in particular suggest that targeting PLK1 with small-molecule inhibitors, in combination with standard of care radiation therapy, will hold a novel strategy in the treatment of DIPG that warrants further investigation. The next step will be to perform detailed in vivo pre-clinical studies to evaluate pharmacokinetic and pharmacodynamic parameters in response to PLK1 inhibition.

## Abbreviations

DIPG: Diffuse Intrinsic Pontine Glioma
PLK1: Polo-like Kinase1
